# Age-related differences in behavioral and brain predictors of parkinsonism in autistic adults

**DOI:** 10.3389/fnagi.2026.1893104

**Published:** 2026-09-29

**Authors:** Manuela Velez Galindo, Goldie A. McQuaid, Rebecca A. Charlton, Edward Ofori, Daniel S. Peterson, David Q. Beversdorf, Melony Valdez, Anthony C. Rodi, Abdullah Sabri, Julia Smith-Plata, Hector Cervantes, Gregory L. Wallace, B. Blair Braden

**Affiliations:** 1 College of Health Solutions, Arizona State University, Phoenix, AZ, United States; 2 Department of Psychology, George Mason University, Fairfax, VA, United States; 3 Department of Psychology, University of London, Goldsmiths, United Kingdom; 4 Thompson Center for Autism & Neurodevelopment, Columbia, MO, United States; 5 Department of Neurology, Radiology & Psychological Sciences, University of Missouri, Columbia, MO, United States; 6 William & Nancy Thompson Endowed Chair in Radiology, Columbia, MO, United States; 7 Department of Speech, Language, and Hearing Sciences, The George Washington University, Washington, DC, United States

**Keywords:** aging, autism spectrum disorder, basal ganglia, motor function, neuroimaging, parkinsonism, repetitive behaviors

## Abstract

Autistic adults experience lifelong motor differences and may have increased vulnerability to parkinsonism, yet behavioral and neuroanatomical correlates of motor challenges across adulthood remain poorly understood. We examined cross-sectional behavioral and motor-related brain region morphology in autistic (*n* = 123) and non-autistic (*n* = 102) adults aged 18–71 years using the Parkinsonism Screening Questionnaire (PSQ), Adult Repetitive Behavior Questionnaire-2 (RBQ-2A), finger tapping, and MRI-derived cortical thickness and subcortical volumes of motor-related brain regions. Replication of the questionnaire analyses was conducted in an independent cohort of middle- and older-aged autistic adults from the Simons Powering Autism Research (SPARK; *n* = 210; ages 42–80 years), whereas structural MRI and finger tapping analyses were limited to the Arizona cohort. Autistic adults reported significantly higher PSQ scores than non-autistic adults. A significant group-by-age interaction was observed for RBQ-2A Factor 1, with autistic adults showing a steeper age-related reduction in repetitive motor and sensory behaviors. RBQ-2A scores were positively correlated with PSQ scores across both cohorts. Finger tapping performance did not differ between groups. Caudate volumes were larger in autistic adults and showed a modestly stronger negative association with age that did not survive multiple comparison correction. These findings suggest that self-reported restricted, repetitive motor behaviors and interests may serve as an indicator of self-reported parkinsonism-like symptoms in autism and highlight the need for longitudinal studies to clarify mechanisms of parkinsonism risk across the lifespan.

## Introduction

Autism spectrum disorder (ASD) is a neurodevelopmental condition characterized by persistent differences in social communication, and the presence of restricted, repetitive behaviors and interests (RRBIs) (American Psychiatric Association, 2013). Although autism research has historically focused on children and young adults, the health and support needs of middle-aged and older autistic adults remain underexplored (Mason et al., 2022). As the population of autistic adults in the United States is estimated to exceed 5.5 million, understanding the effect of aging in this population has become an increasingly important public health priority (Dietz et al., 2020). Many autistic adults rely on aging family members for support, highlighting the need to better understand age-related health trajectories and develop sustainable systems of care as both autistic adults and their caregivers grow older (Piven et al., 2011).

Motor difficulties are a prominent yet often overlooked feature of autism that can substantially affect daily functioning and quality of life (Bhat, 2020; Linke et al., 2020). These challenges include impaired coordination, postural instability, balance difficulties, and reduced grip strength. Motor differences are often among the earliest observable features associated with autism and frequently persist into adulthood, where they may contribute to reduced independence and adaptive functioning (Bhat, 2020; Hau et al., 2022; Landa and Garrett-Mayer, 2006). Emerging evidence suggests that autistic people may be at an elevated risk for parkinsonism and Parkinson’s disease (PD), conditions that profoundly affect movement (Croen et al., 2015; Hand et al., 2020). Parkinsonism refers to a constellation of motor such as bradykinesia, rigidity, tremor, stiffness, and balance instability, that can result from multiple neurological conditions, medications, or PD. On the other hand, PD is a specific neurodegenerative disorder that affects the dopamine producing neurons in the substantia nigra due to the misfolding and aggregation of the protein alpha-synuclein. Healthcare claims and electronic health records show that autistic adults are 6 to 33 times more likely to be diagnosed with PD or related conditions than non-autistic adults (Croen et al., 2015; Hand et al., 2020). In addition, a longitudinal, population-based study from Sweden found increased risk of PD even after controlling for depression, antidepressant use, and antipsychotic exposure in a sample of autistic people who were all less than 50 years of age (Yin et al., 2025). Converging evidence from clinical assessments further supports these findings. A study with two independent samples from Australia and the United States reported high rates of parkinsonism using the Unified Parkinson’s Disease Rating Scale (UPDRS) in autistic adults (Starkstein et al., 2015). Similarly, a subsequent study containing two independent samples of middle age and older autistic adults from the Netherlands and the United States used a self-report screening measure and also found elevated parkinsonism symptoms (Geurts et al., 2022). Despite evidence of increased parkinsonism risk, the behavioral and brain morphology underlying this vulnerability and its relation to other motor features of autism remain unclear.

RRBIs are a core feature of autism and may represent an important behavioral link between autism and parkinsonism since many RRBIs have a prominent motor component. RRBIs include repetitive motor movements, insistence on sameness, and restricted interests (Barrett et al., 2018). Given that parkinsonism is characterized by abnormalities in motor control, repetitive motor behaviors in autism may reflect alterations in shared motor-related brain regions Support for this hypothesis comes from studies of PD, where individuals often exhibit behavioral rigidity and personality traits, including reduced novelty seeking and increased harm avoidance, even before the onset of classical parkinsonian motor symptoms (Poletti and Bonuccelli, 2012). These observations raise the possibility that persistent rigidity-related behaviors in autism may reflect shared motor-related brain morphology with parkinsonism. Understanding the relationship between RRBIs and parkinsonism in autism may provide insights into shared motor-related mechanisms and help identify behavioral markers of parkinsonism in autistic adults.

Neuroanatomical studies have identified structural differences in motor-related brain regions in autistic individuals. Early structural MRI studies frequently reported enlarged caudate volumes in autistic children and young adults (Langen et al., 2007; Sears et al., 1999). More recent neuroimaging studies have continued to identify atypical morphology within motor-related subcortical regions, including the basal ganglia, across autistic adulthood (Christensen et al., 2025; Van Rooij et al., 2018). However, these studies have largely examined broader basal ganglia morphology rather than the caudate and putamen specifically, leaving it unclear whether previously reported caudate differences persist throughout adulthood. This is particularly relevant given that in PD, caudate and putamen volumes typically undergo atrophy, contributing to motor dysfunction (Mak et al., 2015; Pitcher et al., 2012). Understanding whether age-related variations in motor brain regions similar to those observed in PD occur in autistic adults could offer insight into potential risks for parkinsonism. Recent evidence has demonstrated abnormalities in striatal dopamine transporter binding via Single Photon Emission Computed Tomography (SPECT) imaging, an imaging modality used in PD diagnosis, in a significant proportion of young autistic adults (Nuraini et al., 2026). Beyond the basal ganglia, differences in motor-related neurocircuitry extend to the cerebral cortex and cerebellum. Studies have reported significantly thinner precentral and postcentral gyri in autistic adolescents and young adulthood (Hyde et al., 2010; Wallace et al., 2010) and middle adulthood (Scheel et al., 2011) compared to neurotypical individuals. Additionally, alterations in the cerebellum have been linked to both autism and PD (Borghammer et al., 2010; Rojas et al., 2006). In autism, cerebellar abnormalities, such as reduced gray matter volume, have been identified and associated with movement initiation difficulties, impaired motor learning, and reduced executive control of motoric behaviors, paralleling challenges seen in parkinsonism (Becker and Stoodley, 2013; Stoodley, 2016; Yang et al., 2016). Recent research also shows that the cerebellum in autistic individuals exhibits accelerated epigenetic aging (Liu et al., 2023), which may contribute to earlier onset or increased vulnerability to age-related motor difficulties.

Consistent with these structural findings, functional neuroimaging studies suggest that motor network abnormalities in autistic adults persist into older adulthood. Linke et al. (2020) found reduced functional connectivity within the sensorimotor network in middle-aged autistic adults. Similarly, Hau et al. (2022) identified reduced asymmetry in the hand knob area, a region of the precentral gyrus associated with fine motor control, and decreased sensorimotor connectivity in middle-aged and older autistic adults. Complementing these neuroimaging findings, recent behavioral work demonstrates that midlife and older autistic adults show challenges in motor timing and force variability during precision grips tasks (Wang et al., 2025). Collectively, these structural, functional, and behavioral studies suggest that motor system alterations persist throughout adulthood in autism, highlighting the need to determine whether these differences are associated with parkinsonism-related features later in life.

While previous studies have identified elevated parkinsonism features in autistic adults, the relationship with motor challenges, RRBIs, neuroanatomical motor differences, and parkinsonism in aging autistic adults remains poorly understood. This study aimed to address these gaps by investigating behavioral and neuroanatomical correlates of motor function and parkinsonism in middle-aged and older autistic adults using data from an Arizona cohort and a US nation-wide cohort recruited from the Simons Powering Autism Research (SPARK) participant registry. We aimed to: (1) replicate diagnostic group (autistic vs. non-autistic) differences with the hypotheses that autistic adults would report greater parkinsonism features and RRBIs, demonstrate poorer fine motor abilities, and exhibit differences in gray matter characteristics within motor-related brain regions (i.e., larger subcortical volumes and thinner cerebral and cerebellar cortices) compared with non-autistic adults, (2) evaluate the hypothesis that autistic adults demonstrate stronger positive age associations with parkinsonism features and RRBIs, and stronger negative age associations with fine motor abilities and gray matter characteristics of motor-related brain regions, and (3) evaluate the hypothesis that behavioral and brain measures are associated with self-reported parkinsonism in autistic adults across the lifespan.

## Methods

### Participants

#### Cohort 1: Arizona

Autistic participants were recruited through a community partnership with Southwest Autism Research and Resource Center (SARRC), which maintains a database of individuals across the lifespan who consented to be contacted for future research studies. Additional participants were recruited through flyers distributed throughout Tempe, Arizona, Arizona State University (ASU) community events. Non-autistic participants were recruited through community flyers, media releases and advertisements through ASU, the Banner Alzheimer’s Prevention Registry, and word of mouth. All autistic participants had their diagnosis formally verified at SARRC using the Autism Diagnostic Observation Schedule-2 (ADOS-2) Module 4 (Lord et al., 2012), along with a brief psychiatric history interview and, when needed, the Autism Diagnostic Interview-Revised. A licensed psychologist with over 25 years of diagnostic experience confirmed that all autistic participants met DSM-5 criteria. Additional inclusion criteria for the autistic group included a T-score above 65 on the self-report Social Responsiveness Scale-2 (SRS-2). Inclusion criteria for the non-autistic group were: (1) no self-reported suspected or established diagnosis of ASD, (2) a T-score below 65 on the self-report SRS-2, and (3) no first-degree relative with an autism diagnosis. General inclusion criteria for all participants included: (1) a score above 70 on the Kaufman Brief Intelligence Test-2 (KBIT-2), (2) a score above 25 on the Mini-Mental State Examination (MMSE), and (3) no neurological conditions, serious mental illness that interfered with study participation, or known genetic disorders affecting brain and behavior. Self-reported years of education were recorded. The final Arizona sample consisted of 225 adults (autistic: *n* = 123; non-autistic: *n* = 102) between 18 and 71 years of age (see Table 1 for participant demographics). The study was approved by ASU’s Institutional Review Board (IRB) STUDY 00020130 and all participants provided informed consent.

**TABLE 1:** Demographics of the Arizona and SPARK cohorts.

Demographics	Cohort		
	Arizona		SPARK
	Autistic *n* = 123 (54.7%)	Non-autistic *n* = 102 (45.3%)	Autistic *n* = 210 (100%)
Age – mean (SD) and range	40.85 (15.97), 18–71	41.92 (16.35) 18–70	54.9 (9.3), 42–80
Sex – n (%)			
Male	76 (61.8%)	60 (58.8%)	89 (42.4%)
Female	47 (38.2%)	42 (41.2%)	121 (57.6%)
ADOS-2 – mean (SD) and range	10.37 (2.83), 7–19	–	–
IQ – mean (SD) and range	108.17 (14.84), 70–148	107.83 (11.45), 77–141	–
SRS-2 – mean (SD) and range	76.67 (6.91), 64–90	45.44 (5.93), 37–64	–
Race – n (%)			
White	84 (68.3%)	64 (62.7%)	175 (83.3%)
Black	4 (3.3%)	2 (2.0%)	3 (1.4%)
Asian	5 (4.1%)	3 (2.9%)	6 (2.9%)
Native American/Alaska Native	0 (0.0%)	1 (1.0%)	3 (1.4%)
More than one race	0 (0.0%)	0 (0.0%)	17 (8.1%)
Other	1 (0.8%)	1 (1.0%)	6 (2.9%)
Missing	29 (23.6%)	31 (30.4%)	–
Ethnicity – n (%)			
Latino/a/x	7 (5.6%)	11 (10.8%)	8 (3.8%)
Unknown	0 (0.0%)	0 (0.0%)	2 (1.0%)
Not Latino/a/x	116 (94.3%)	91 (89.2%)	198 (94.3%)
Missing	0 (0.0%)	0 (0.0%)	2 (1.0%)
Education – n (%)			
Bachelor’s degree or higher	53 (43.1%)	63 (61.8%)	137 (65.2%)
Less than Bachelor’s degree	70 (56.9%)	38 (37.3%)	72 (34.3%)
Missing	0 (0.0%)	1 (1.0%)	1 (0.5%)
Current antipsychotic use – n (%)			
Yes	7 (5.7%)	0 (0%)	30 (14.3%)
No	116 (94.3%)	102 (100%)	180 (85.7%)

Values are presented as mean (SD), range, or n (%) as indicated. ADOS-2, Autism Diagnostic Observation Schedule-2; IQ, intelligence quotient; SPARK, Simons Powering Autism Research for Knowledge; SRS-2, Social Responsiveness Scale-2. En dashes indicate that the measures was not available for the Arizona Non-autistic participants or SPARK cohort.

#### Cohort 2: Simons power autism research for knowledge (SPARK)

Participants were recruited online through the Simons Powering Autism Research for Knowledge (SPARK) Research Match service as part of a longitudinal study of adulthood and aging in autism (RM0135Wallace_Adult). All participants in the current study were designated by SPARK as “independent” autistic adults, indicating that they were able to provide informed consent independently. SPARK requires participants to self-report an ASD diagnosis made by a medical or clinical professional (which may or may not have included an ADOS-2 or Autism Diagnostic Interview Revised [ADI-R]). Autism diagnoses within the SPARK cohort have been validated through comparison with electronic medical records, with 98.8% of reported diagnoses confirmed (Fombonne et al., 2022). In the current study, 99% of participants scored above the cutoff (>65) on the short form of the Autism Quotient, further supporting the presence of ASD. Educational attainment was collected based on participants reporting either less than a bachelor’s degree or a bachelor’s degree or higher. The final SPARK sample consisted of 210 autistic adults between 42 and 80 years of age (89 males, 121 females; see Table 1 for participant demographics). Taken together, there is a high confidence that autistic participants from SPARK have valid autism diagnoses despite lack of additional ADOS-2 assessments for this specific study. The study was approved by The George Washington University IRB and all participants provided informed consent.

### Assessment overview

Participants from the Arizona cohort completed the Parkinsonism Screening Questionnaire (PSQ), Adult Repetitive Behaviors Questionnaire-2 (RBQ-2A), finger tapping assessment, and structural MRI, although availability varied across assessments (See Supplementary Table). Participants from the SPARK cohort completed the PSQ and RBQ-2A but did not undergo finger tapping assessment or structural MRI.

### Parkinsonism screening questionnaire (PSQ)

PSQ data were available for 91 participants in the Arizona cohort (autistic: *n* = 41; non-autistic: *n* = 49), while 134 participants did not have PSQ data available and were therefore not included in PSQ analyses. In the SPARK cohort, PSQ data were available for 205 autistic participants. The PSQ is a six-item self-report measure developed to screen for parkinsonism in population-based studies (Fereshtehnejad et al., 2014; Geurts et al., 2022). It assesses stiffness, tremors, difficulty with buttoning and dressing, reduced arm swing while walking, a sensation of being “stuck” when initiating movement or changing direction, and overall decline in motor function. Responses included “Yes,” “No,” or “I don’t know.” “Yes” responses were scored as 1, while “No” and “I don’t know” responses were scored as 0, resulting in total scores ranging from 0 to 6. Higher total scores indicate more self-reported parkinsonism symptoms.

### Adult Restricted Behaviors Questionnaire-2 (RBQ-2A)

The self-report Adult Repetitive Behaviors Questionnaire-2 (RBQ-2A) was completed by 176 participants in the Arizona cohort (autistic: *n* = 96; non-autistic: *n* = 80); participants without available RBQ-2A data (*n* = 49) were not included in the analyses. In the SPARK cohort, RBQ-2A data were available for 210 autistic participants. The same number of participants contributed to the Total, Factor 1, and Factor 2 analyses. The RBQ-2A is comprised of 20 items, scored on either a 3- or 4-point scale. To allow for consistent comparisons across repetitive behaviors and their sub-factors, responses rated as ‘4’ were recoded as ‘3’. The Total Mean Score is calculated by summing each participant’s item scores (American Psychiatric Association, 2013; Dietz et al., 2020; Mason et al., 2022) across all 20 items and dividing this total by the number of items completed. The resulting value represents a total mean score ranging from 1 to 3 (Barrett et al., 2018). The RBQ-2A includes two empirically derived subscales based on the factor structure: Factor 1: Repetitive Sensory-Motor Behavior (items 2, 3, 4, 5, 6, 10) and Factor 2: Insistence on Sameness (items 1, 7, 11–19) (Barrett et al., 2018). Factor 1 reflects repetitive motor movements and sensory-driven behaviors (e.g., stereotyped movements and sensory seeking) (Barrett et al., 2018). In contrast, Factor 2 reflects cognitive-behavioral rigidity, including resistance to change, strong adherence to routines, compulsive behaviors. Items 8 and 9 are not included in either subscale analyses (Barrett et al., 2018). Mean subscale scores were calculated by averaging item responses (rated 1–3) within each subscale, yielding scores on the same 1-3 scale (Barrett et al., 2018). Higher total and subscale scores indicate greater severity of restricted and repetitive behaviors.

### Finger tapping

Finger tapping performance was assessed in 200 participants from the Arizona cohort (autistic: *n* = 107; non-autistic: *n* = 93) using the finger tapping oscillation assessment. Participants without available finger tapping data (*n* = 25) were not included in the analyses. Using their index finger, starting with their dominant hand, participants completed three trials with each hand, resulting in a total of six trials. Each participant performed the task in seated position with their forearms resting on the table, maintaining a flat hand, wrist, and finger position throughout the task. Participants were asked to tap the metal bar as quickly as possible for 10 s. Performance was calculated as the average number of taps across the three trials for each hand. Higher finger tapping scores indicate better motor performance, while lower scores indicate poorer motor performance.

### MRI and motor brain regions

Structural MRI was acquired only in the Arizona cohort. High-resolution structural brain images were obtained from 205 Arizona participants (autistic: *n* = 111; non-autistic: *n* = 94). Participants without MRI data (*n* = 20) were not included in the analyses. MRI data were acquired at Barrow Neurological Institute using a 3-Tesla Philips Ingenia MRI scanner with a maximum gradient strength of 45 mT/m. T1-weighted images were acquired using a 3D magnetization-prepared rapid acquisition gradient echo (MPRAGE) sequence with the following parameters: repetition time (TR) = 6.744 ms, echo time (TE) = 3.106 ms, flip angle = 9°, voxel size = 1.05 × 1.05 × 1.20 mm, 256 × 256 in-plane resolution, a 240 mm field of view, and 170 sagittal slices with slice thickness of 1.2 mm. T1-weighted images were processed using FreeSurfer to reconstruct cortical surface models and calculate cortical thickness as the distance (in millimeters) between the gray-white matter boundary and the pial surface (Fischl and Dale, 2000). Quality control of all FreeSurfer outputs was performed according to Qoala-T and the Enhancing Neuro Imaging Genetics through Meta-Analysis (ENIGMA) structural MRI quality control protocol (ENGIMA, 2017; Klapwijk et al., 2019), including visual inspection of cortical surface reconstructions and subcortical segmentation to identify reconstruction or segmentation errors prior to statistical analyses (ENGIMA, 2017; Klapwijk et al., 2019). All MRI scans included in the final imaging sample passed quality control. FreeSurfer provides a robust and widely recognized approach for quantifying both cortical thickness and subcortical as well as cerebellar volumes (Han et al., 2006) Cortical thickness was extracted for the left and right precentral gyri. Gray matter volumes were calculated for caudate, putamen, and cerebellum. All regional gray matter volumes were normalized to estimated total intracranial volume (eTIV) by dividing each raw regional volume by eTIV and multiplying by 100 to obtain a percentage of eTIV.

### Statistical analyses

Analyses were conducted in IBM SPSS Statistics (Version 29.0.2.0). For the Arizona sample, we used multiple regression to evaluate diagnosis group differences and diagnosis group-by-age interactions, with age, sex, and education included as covariates, for all brain and behavior measures of interest. For both samples, correlations were conducted to investigate the association between PSQ scores and brain and behavior measures in autistic participants, adjusting for age, sex, and education [Arizona cohort: continuous years of education; SPARK cohort: dichotomous less than a bachelor’s degree vs. bachelor’s degree or higher (Hayward et al., 2019)]. To control for multiple comparisons within hypothesized domains and regions of interest, p values were adjusted using Benjamini-Hochberg false discovery rate (FDR) within predefined families of related outcome measures. Specifically, behavioral outcomes were corrected within multiple scores from the same measurement tool (e.g., RBQ-2A total, Factor 1, and Factor 2) and neuroimaging measures were corrected for comparisons across the two hemispheres (e.g., left and right caudate volume). PSQ analyses were not corrected since there was only one measures from this hypothesis-driven instrument. For both samples, exploratory correlations between PSQ scores and age were evaluated within diagnosis groups, regardless of the presence of a significant diagnosis group-by-age interaction because of the current lack of information regarding parkinsonism in autistic adults across the lifespan. Alpha was set at 0.05.

## Results

### Behavioral outcomes

#### PSQ

##### Arizona cohort

There was a significant main effect of diagnostic group with the autistic group demonstrating higher PSQ scores (i.e., more self-reported parkinsonism symptoms) than the non-autistic group, when controlling for age, sex, and education [β = 0.33, t(84) = 3.24, *p* = 0.002; Figure 1A]. However, the interaction between diagnostic group and age was not significant for PSQ scores [β = −0.14, t(83) = −0.41, *p* = 0.68; Figure 1B]. Exploratory correlations showed that age and PSQ were not correlated in the autistic group [r(35) = 0.09, *p* = 0.57; Figure 1B], when controlling for sex and education.

**FIGURE 1:**
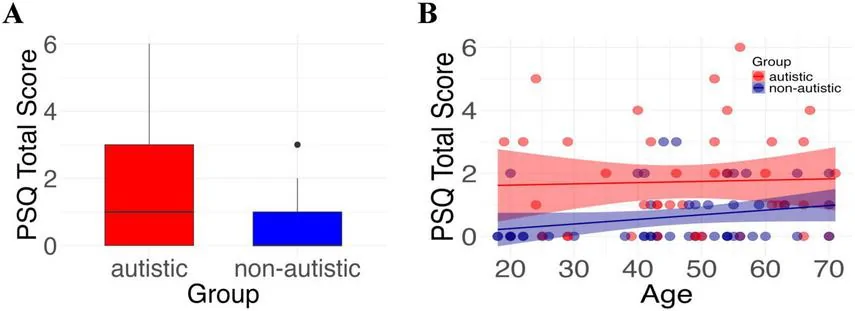
Parkinsonism symptom severity by diagnostic group across age in Arizona cohort. **(A)** PSQ total score by diagnostic group. **(B)** PSQ total scores across age in autistic and non-autistic adults. PSQ, Parkinsonism Screening Questionnaire.

##### SPARK cohort

In the SPARK cohort of autistic adults, age also did not correlate with PSQ scores [r(205) = 0.08, *p* = 0.22], when controlling for sex and education.

#### RBQ-2A

##### Arizona cohort

The autistic group had a higher RBQ-2A scores than the non-autistic group on the total score [β = 0.85, t(167) = 5.11, uncorrected *p* = 0.001, FDR-corrected *p* = 0.001], Factor 1 [β = 0.90, t(167) = 5.00, uncorrected *p* = 0.001, FDR-corrected *p* = 0.001: Figure 2A], and Factor 2 [β = 0.79, t(167) = 4.79, uncorrected *p* = 0.001, FDR-corrected *p* = 0.001], when controlling for age, sex, and education. A significant group-by-age interaction was found for RBQ-2A Factor 1 scores, indicating that the relationship between age and Factor 1 differed across autistic and non-autistic groups (β = −0.46, t(167) = −2.40, uncorrected *p* = 0.01; FDR-corrected *p* = 0.05: Figure 2A), when controlling for age, sex, and education. Specifically, age was negatively correlated with RBQ-2A Factor 1 scores in the autistic group with a small effect size (r(89) = −0.28, *p* = 0.006: Figure 2A), but not in the non-autistic group (r(72) = 0.01, *p* = 0.81), when controlling for sex and education. In contrast, no significant associations with age or group-by-age interactions were observed for RBQ-2A Factor 2 (age: r(94) = −0.06, *p* = 0.56; age x group: β = −0.16, t(167) = −0.92, *p* = 0.35) or Total Scores (r(94) = −0.14, *p* = 0.16; age x group: β = −0.24, t(167) = −1.36, *p* = 0.17).

**FIGURE 2:**
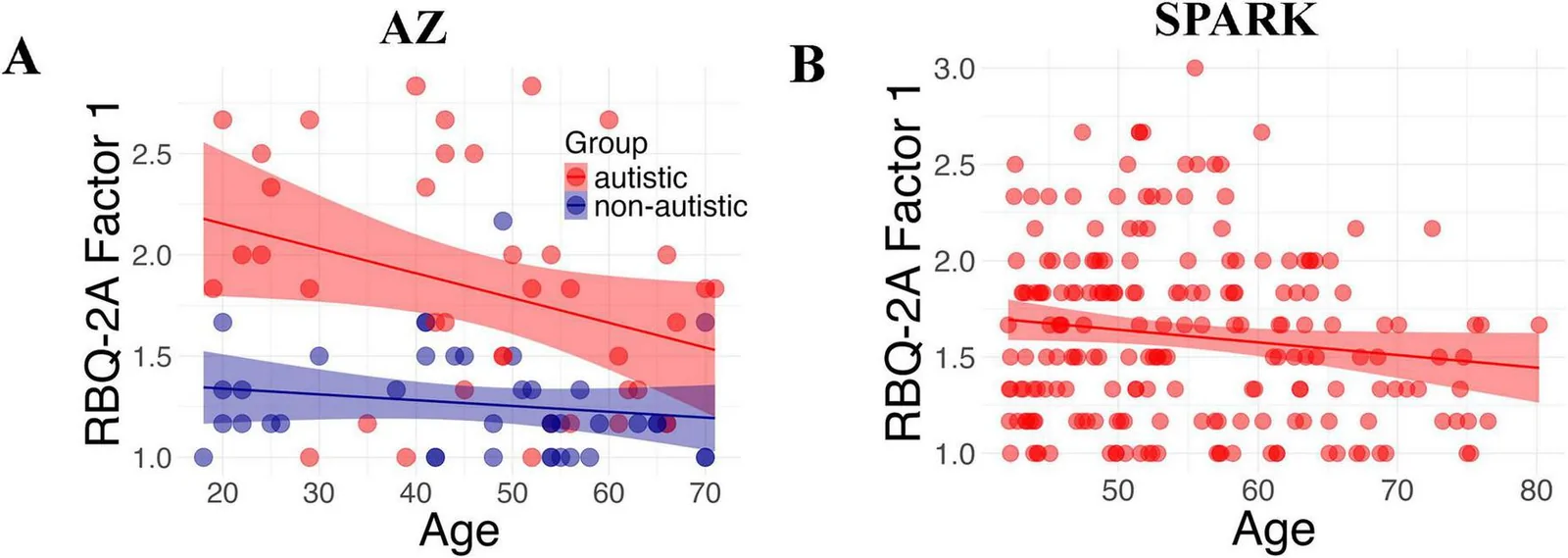
Age relationships with RBQ-2A Factor 1 scores in **(A)** autistic and non-autistic adults in the Arizona cohort and **(B)** autistic adults in the SPARK cohort. RBQ-2A, Repetitive Behavior Questionnaire-2 Adult.

##### SPARK cohort

Age was not significantly associated with RBQ-2A Factor 1 scores (r(209) = −0.12, uncorrected *p* = 0.04, FDR-corrected *p* = 0.20; Figure 2B) after controlling for sex and education. Similarly, no significant correlations were found between age and RBQ-2A Factor 2 scores (r(209) = 0.03, uncorrected *p* = 0.61, FDR-corrected *p* = 0.78) or RBQ-2A Total Score (r(209) = −0.01, uncorrected *p* = 0.78, FDR-corrected *p* = 0.78), after adjusting for sex and education.

#### Finger tapping

##### Arizona cohort only

There were no group differences or group by age interactions for left-hand (group:β = −0.13, t(192) = −0.70, *p* = 0.48; age x group: β = 0.14, t(192) = 0.73, *p* = 0.46) or right-hand tapping performance (group:β = −0.06, t(192) = −0.35, *p* = 0.72; age x group: β = 0.07, t(192) = −0.39, *p* = 0.69), when controlling for age, sex, and education.

##### Structural MRI – Arizona cohort only

For left caudate volumes, diagnostic group was a significant predictor, with the autistic group showing larger volumes than the non-autistic group, when controlling for age, sex and education (β = 0.42, t(196) = 2.47, uncorrected *p* = 0.01, FDR-corrected *p* = 0.02; Figure 3A). A group-by-age interaction was also observed (β = −0.36, t(196) = –2.02, uncorrected *p* = 0.04, FDR-corrected *p* = 0.06; Figure 3A), when controlling for age, sex, and education, such that the autistic group showed a steeper negative association between age and left caudate volume (r(110) = –0.43, *p* < 0.001), compared to the non-autistic group (r(90) = –0.30, *p* = 0.003). However, this interaction did not survive FDR correction.

**FIGURE 3:**
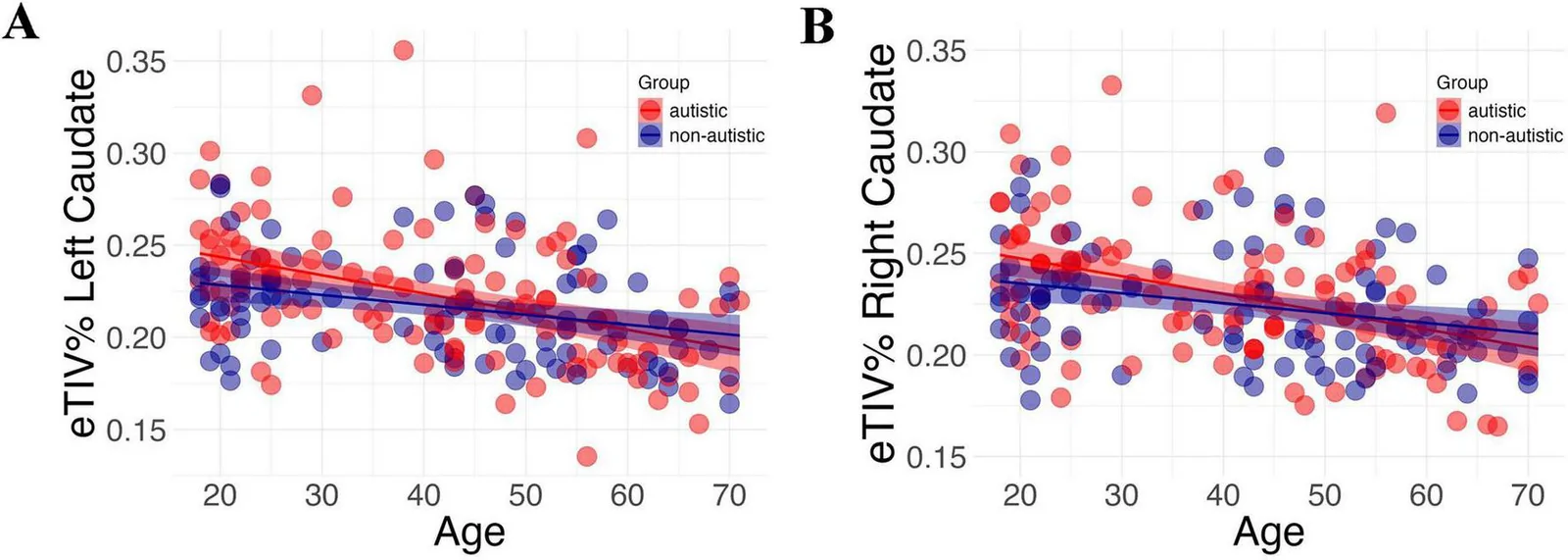
eTIV-adjusted left and right caudate volume by diagnostic group across age in Arizona cohort **(A)** eTIV% left caudate volume and **(B)** eTIV% right caudate volume. eTIV, estimated total intracranial volume.

Similarly, diagnostic group was a significant predictor of right caudate volume, with the autistic group showing larger right caudate volumes than the non-autistic group, when controlling for age, sex, and education (β = 0.39, t(196) = 2.24, uncorrected *p* = 0.02, FDR-corrected *p* = 0.02; Figure 3B). However, the group-by-age interaction approached, but did not reach significance (β = −0.34, t(196) = –1.86, uncorrected *p* = 0.06, FDR-corrected *p* = 0.06; Figure 3B, when controlling for age, sex, and education. There were no significant effects in the precentral gyrus, putamen, or cerebellar cortex.

#### PSQ correlations with RBQ-2A

##### Arizona cohort

Correlations revealed that RBQ-2A Factor 2 (r(35) = 0.42, uncorrected *p* = 0.009, FDR-corrected *p* = 0.01: Figure 4B), and Total Score (r(35) = 0.44, uncorrected *p* = 0.007; FDR-corrected *p* = 0.01: Figure 4C) were positively correlated with the PSQ scores in the autistic group, showing small to moderate effect sizes after adjusting for age, sex, and education. However, Factor 1 approached but did not reach significance (r(35) = 0.30, uncorrected *p* = 0.06, FDR-corrected *p* = 0.06; Figure 4A) after adjusting for age, sex, and education.

**FIGURE 4:**
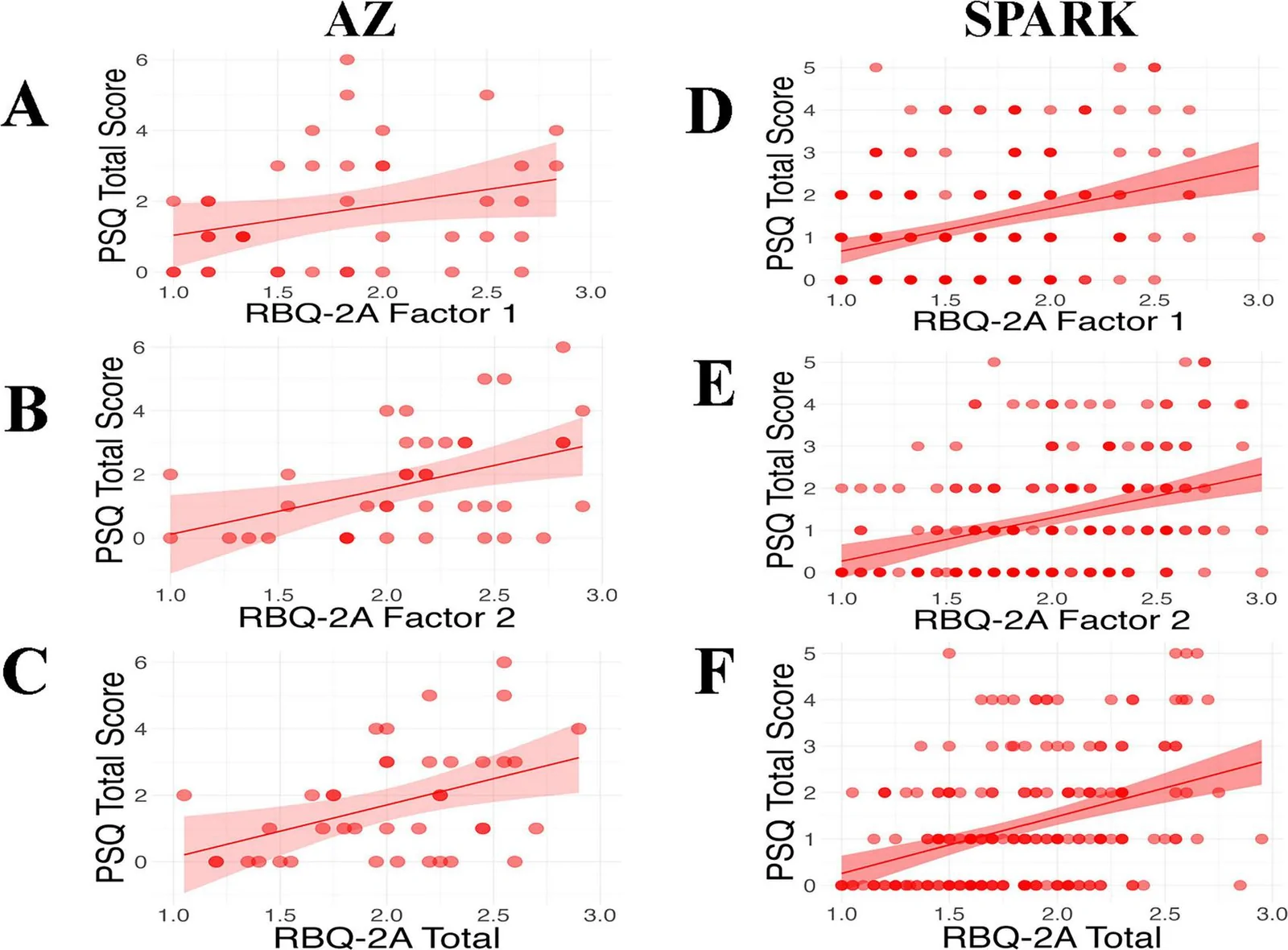
Association between RBQ-2A scores and parkinsonism symptoms in Arizona and SPARK cohorts. All panels display autistic participants only. **(A–C)** RBQ-2A Factor 2, and Total scores and PSQ total scores in the Arizona autistic group. **(D–F)** RBQ-2A Factor 1, Factor 2, and Total scores and PSQ total scores in the SPARK autistic group. RBQ-2A, Repetitive Behavior Questionnaire-2 Adult; PSQ, Parkinsonism Screening Questionnaire.

##### SPARK cohort

Correlations revealed that the RBQ-2A Factor 1 (*r*(204) = 0.33, uncorrected *p* < 0.001, FDR-corrected *p* < 0.001; Figure 4C, Factor 2 (*r* (204) = 0.36, uncorrected *p* < 0.001, FDR-corrected *p* < 0.001; Figure 4D), and Total Score (r (204) = 0.38, uncorrected *p* < 0.001, FDR-corrected *p* < 0.001; Figure 4E) were positively correlated with the PSQ scores in the SPARK cohort, all with medium effect sizes after adjusted for age, sex, and education.

##### PSQ correlations with structural MRI – Arizona cohort only

There were no significant correlations between PSQ scores and left and right caudate volumes. However, due to the explicit role of the caudate in motor function, correlations were explored with Factor 1 (Repetitive Motor Movements) of the RBQ-2A. The TIV% was not adjusted for these within-participant analyses. The left and right caudate volumes positively correlated with RBQ-2A Factor 1 scores, with small-to-medium effect sizes (Left: (r(82) = 0.25, uncorrected *p* = 0.01; Figure 5A; Right: (r(82) = 0.23, uncorrected *p* = 0.02; Figure 5B). In addition, there were no associations between left and right caudate for RBQ-2A Factor 2 and Total Mean Scores.

**FIGURE 5:**
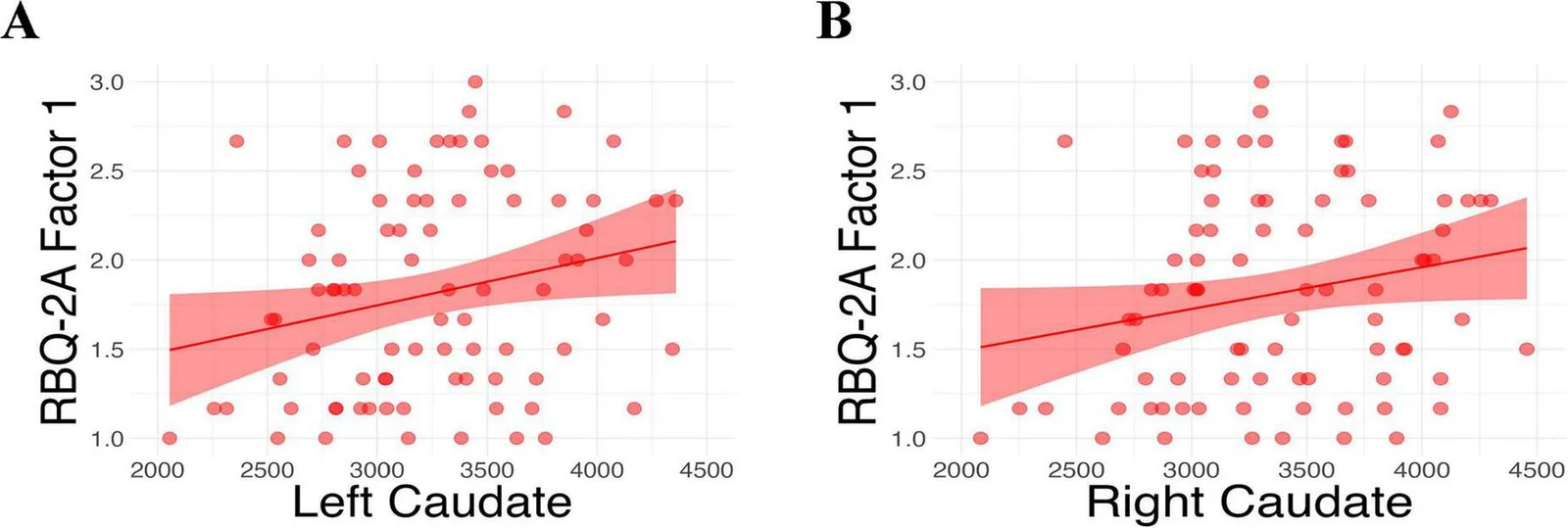
Association between **(A)** left and **(B)** right caudate volume and RBQ-2A Factor 1 (repetitive–motor sensory behaviors) in autistic adults. RBQ-2A, Repetitive Behavior Questionnaire-2 Adults.

##### Finger tapping performance – Arizona cohort only

Left- and right-hand finger tapping performance were negatively, but not significantly, associated with PSQ scores after controlling for age, sex, and education (left:(r(31) = −0.18, uncorrected *p* = 0.28; FDR-corrected *p* = 0.28; right: *r*(31) = −0.19, uncorrected *p* = 0.25; FDR-corrected *p* = 0.28).

## Discussion

This study evaluated age-related differences in behavioral and brain motor features and relationships with parkinsonism in autistic adults using two independent cohorts when possible. Consistent with our hypotheses and prior research, autistic adults self-reported significantly higher parkinsonism symptoms than non-autistic adults, replicating findings from previous population-based and clinical studies (Croen et al., 2015; Geurts et al., 2022; Hand et al., 2020; Starkstein et al., 2015). Across both cohorts, correlation analyses identified positive associations between self-reported RRBIs and self-reported parkinsonism symptoms. However, because both the PSQ and RBQ-2A rely on self-report and these analyses were cross-sectional, these findings should be interpreted cautiously. Neuroanatomically, we observed larger caudate volumes in autistic adults compared to non-autistic peers, consistent with longstanding findings in younger populations (Estes et al., 2011; Langen et al., 2007; Stanfield et al., 2008). We also identified a novel pattern of potentially steeper negative age relationship with left caudate volume among autistic adults, albeit with a small effect size that did not survive correction for multiple comparisons. However, contrary to our hypotheses, caudate volume was not directly associated with parkinsonism symptom severity, though it did correlate with repetitive motor behaviors as seen in other studies (Estes et al., 2011; Langen et al., 2014). Together, these findings replicated elevated self-reported parkinsonism symptoms in autistic adults and provide new evidence linking self-reported RRBIs and with self-reported parkinsonism symptoms, while suggesting that caudate morphology is more closely related to repetitive motor behaviors than parkinsonism symptom severity.

The associations between RRBIs and parkinsonism-like symptoms observed across both cohorts are novel findings, although their interpretation is limited by the self-report and cross-sectional nature of these analyses. While the present findings do not establish a shared underlying neurobiological mechanism, prior research provides a rationale for considering whether overlapping motor-related pathways may contribute to these behavioral associations. Specifically, structural and functional abnormalities in the basal ganglia, particularly the caudate nucleus, have been consistently associated with the presence and severity of repetitive behaviors in autistic individuals (Langen et al., 2014; Linke et al., 2020; Turner et al., 2006). Our findings of a positive relationship between caudate size and repetitive motor aspects of RRBIs align with this literature. However, these exploratory analyses were not adjusted for covariates or multiple comparisons, so should be interpreted cautiously. This is broadly consistent with well-established basal ganglia findings in PD where degeneration of these pathways, especially those involved in movement initiation and motor flexibility, leads to hallmark motor systems such as rigidity, bradykinesia, and tremor (DeLong and Wichmann, 2007; Wu et al., 2015). One possible explanation is that both RRBIs and parkinsonism symptoms may involve overlapping cortico-striatal pathways responsible for motor planning, execution, and inhibition, although the present findings do not directly demonstrate shared neurobiological mechanisms (DeLong and Wichmann, 2007; Obeso et al., 2008). In autism, these circuits are likely altered due to early neurodevelopmental differences, whereas in PD, similar circuits become impacted through progressive dopaminergic degeneration. This shared reliance on basal ganglia-mediated motor control may in part explain why autistic adults with higher levels of RRBIs are more likely to report parkinsonism symptoms. Notably, recent work in middle-aged and older autistic adults has shown that temporal features of motor control are significantly associated with repetitive behaviors, even in the absence of a PD diagnosis, suggesting a relationship between motor control and core autistic traits across adulthood (Wang et al., 2025). Repetitive non-tremor motor movements have also been documented in PD, including stereotyped or perseverative movements distinct from classical tremor (Kurlan, 2004; Pallanti et al., 2010; Rajalingam and Fasano, 2023). Together, these observations highlight that repetitive motor phenomena are not unique to autism but can also emerge in neurodegenerative conditions affecting basal ganglia circuits. This convergence is consistent with the possibility that overlapping motor-related pathways contribute to repetitive motor behaviors across conditions, although the underlying biological mechanisms are likely distinct between neurodevelopmental and neurodegenerative disorders.

In this study, we did not observe significant group differences in finger tapping performance or group by age interactions. Although a nonsignificant trend was observed between slower dominant-hand finger tapping and higher self-reported parkinsonism symptoms in autistic adults, fine motor performance may reflect individual variation in motor symptoms among autistic adults rather than a general feature of autism or an autism-specific marker of age-related decline. Fine motor slowing, or bradykinesia, is a cardinal symptom and an early clinical marker of PD progression (Berardelli, 2001; Kang et al., 2011). Finger tapping speed is widely used in PD for assessing motor slowing and rigidity, often sensitive to even subtle declines in motor function (Goetz et al., 2008). Recent behavioral work demonstrates that fine motor control differences persist into midlife and older adulthood in autism, with aging autistic adults showing group differences in motor timing and variability (Wang et al., 2025). While fine motor difficulties are well-documented in autistic children, including reduced manual dexterity, slower motor execution, and impaired coordination, there is a notable lack of research examining these issues in autistic adults (Fournier et al., 2010; Linke et al., 2020). Existing studies suggest that motor difficulties, including fine motor challenges, persist into adulthood in autism (Travers et al., 2017), but little is known about how these difficulties evolve with age or how they relate to parkinsonism-like symptoms. Longitudinal work incorporating objective motor and clinician-rated assessments is needed to determine whether fine motor differences are associated with changes in parkinsonism symptoms over time.

Our finding of larger caudate volumes in autistic adults compared to non-autistic adults is consistent with longstanding literature documenting caudate enlargement in autistic children and adolescents (Estes et al., 2011; Langen et al., 2007; Sears et al., 1999). This pattern has been widely interpreted as a neurodevelopmental feature of autism, particularly because caudate enlargement has been linked to the presence and severity of repetitive motor behaviors (Estes et al., 2011; Qiu et al., 2016; Turner et al., 2006). Our findings suggest that larger caudate volumes are present in autistic adults, extending previous observations of caudate enlargement reported in younger autistic populations. Although we observed a novel pattern of potentially steeper negative age relationship with left caudate volume among autistic adults, this did not survive correction for multiple comparisons and therefore should be interpreted cautiously. This pattern is broadly consistent with emerging lifespan neuroimaging work. For example, Koolschijn and Geurts (2016) reported robust age-related gray matter decline in autistic adults but found no group differences in subcortical volumes, suggesting that age may overshadow early-life enlargement in later adulthood. Similarly, Van Rooij et al. (2018) found no significant age by diagnosis group interactions in subcortical structures, including the caudate, across a large sample spanning childhood to adulthood. Christensen et al. (2025) also did not observe significant age by group effects reported in basal ganglia volumes; however, visually the oldest autistic adults appear to have smaller basal ganglia compared to controls. Their basal ganglia composite included regions such as the putamen and pallidum, which may follow distinct developmental trajectories, and therefore, region-specific effects within the caudate may have been diluted. Consistent with prior work, caudate volume was associated with sensorimotor behaviors (Estes et al., 2011; Stanfield et al., 2008) but not directly related to parkinsonism symptom severity in this sample. This dissociation suggests that caudate morphology may be more closely related to core autism-related motor traits than parkinsonism features.

We did not observe significant group differences in the precentral gyrus, putamen, or cerebellar cortex, indicating that morphology differences are not widespread across motor-related brain regions. This stands in partial contrast to several previous studies reporting reduced precentral gyrus thickness in autistic adolescents and adults (Hyde et al., 2010; Scheel et al., 2011), reduced cerebellar volumes (Rojas et al., 2006) or putamen differences (Sato et al., 2014). However, the literature on motor-related neuroanatomical differences in autism is mixed, with some studies failing to replicate findings, particularly in adult samples (Ecker, 2012; Haar et al., 2016). Several factors may account for these discrepancies. First, many prior studies have focused on children and adolescents, whereas our sample included a wider adult age range, where neurodevelopmental differences may diminish or become less detectable relative to developmental cohorts. Second, methodological differences, including imaging protocols, cortical parcellation approaches, and statistical analyses, may influence the detection of subtle morphometric differences. Third, sample characteristics, such as sex distribution or overall sample size could contribute to variability across studies. Finally, it is possible that age-related motor vulnerability in autism is less dependent on structural differences in regions such as the precentral gyrus, putamen, or cerebellum, and may instead reflect functional disruptions in motor networks or neurochemical changes, possibilities that warrant further investigation.

Surprisingly, PSQ scores were not positively associated with age within the autistic group (exploratory analyses), as is common amongst the general population, which underscores the need for longitudinal research to distinguish neurodevelopmental traits from aging-related changes. Although parkinsonism symptoms are often expected to increase with age, particularly when arising from PD, these cross-sectional findings should be interpreted cautiously. Understanding the true aging trajectory of parkinsonism symptoms in autistic adults will require longitudinal research that controls for factors such as cohort effects, individual variability, and heterogeneous biological mechanisms. The biological mechanisms underlying the self-reported parkinsonism symptoms observed in autistic adults remain unknown and cannot be determined from the present study. The presence of these symptoms across autistic adulthood, including in younger adults, raises questions about whether they reflect mechanisms distinct from or overlapping with age-related PD processes. Biomarker, pharmacological, and post-mortem neuropathological research is needed to understand the underlying mechanism(s) of parkinsonism in autistic adults. Interestingly, RBQ-2A Factor 1 was negatively associated with age in autistic adults across both cohorts, which is consistent with previous research (Barrett et al., 2018), but this relationship did not survive multiple comparison correction in the SPARK cohort. Factor 1 was significantly associated with PSQ scores in the SPARK cohort but did not reach statistical significance in the Arizona cohort. Longitudinal research is needed to fully evaluate the aging trajectories of repetitive sensory-motor behaviors and parkinsonism symptoms in autistic adults.

There are several limitations that should be considered when interpreting these findings. First, the cross-sectional design precludes conclusions about causality or developmental/aging progression. Longitudinal studies will be necessary to determine whether motor symptoms and neuroanatomical differences reflect stable characteristics, age-related change, or emerging neurodegenerative processes. Second, parkinsonism symptoms were assessed via self-report using the PSQ, which has not been clinically validated against formal neurological examinations or diagnostic criteria for PD, and autistic adults may experience motor symptoms differently due to differences in interoceptive awareness. As a result, some motor limitations may be under- or over-reported depending on how changes are perceived relative to an individual’s typical functioning, highlighting the importance of future studies incorporating objective motor assessments and clinician-rated measures. As such, the findings reflect subjective reports of parkinsonism-like symptoms rather than confirmed clinical diagnoses. The RBQ-2A also relies on self-report, and therefore their associations reflect correspondence between self-reported behavioral features rather than evidence of predictive relationships or shared underlying neurobiological mechanisms. The present findings should not be interpreted as evidence of PD or as indicating that individuals reporting greater RRBIs or parkinsonism symptoms are more likely to subsequently develop PD. Longitudinal studies incorporating objective motor measures and clinician-rated assessments will be necessary to determine whether these behavioral features are associated with subsequently developing clinically diagnosed PD. Third, IQ data was not included as a covariate in the primary analyses. In the Arizona cohort, IQ did not significantly differ between diagnostic groups, and when included in the regression models, it did not explain significant additional variance. Additionally, IQ data were not available for the SPARK cohort, preventing consistent adjustments for IQ across both cohorts. However, future studies should more fully evaluate the role of IQ in parkinsonism and related behavioral and brain features. Fourth, neuroimaging and finger tapping data were only available for the Arizona cohort, limiting the generalizability of these findings. Finally, we did not include autistic individuals with co-occurring intellectual disability, which limits the generalizability of these findings to the full autism spectrum. We also did not examine potential sex differences, which may be relevant given evidence of sex-based variability in both autism neurobiology and parkinsonism risk and are warranted in future studies.

This study provides further evidence of self-reported parkinsonism features in autistic adults and demonstrated that these features are behaviorally associated with self-reported RRBIs but not with caudate volume. These findings suggest that self-reported RRBIs may represent behavioral correlates of self-reported parkinsonism in autistic adults and warrant further investigation as potential markers of motor vulnerability. Understanding these brain and behavior correlates of parkinsonism in autistic adults is critical for supporting the health and well-being of the growing population, a group historically underrepresented in both autism and aging research.

## Data Availability

The datasets presented in this article are not readily available because the SPARK data are available through the SPARK repository. Arizona cohort data are partially available through NIMH Data Archive, and remaining data may be made available from the corresponding author upon reasonable request. Requests to access the datasets should be directed to BB, bbbraden@asu.edu.
