# Supplementary material for: Age-related differences in behavioral and brain predictors of parkinsonism in autistic adults
Source: Front Aging Neurosci. 2026 Sep 29;18:1893104. doi: 10.3389/fnagi.2026.1893104 (PMC13658442; doi:10.3389/fnagi.2026.1893104)
Supplement: Supplementary file 1 [file Data_Sheet_1.pdf]

Supplementary Table. Behavioral and neuroimaging measures

	Arizona Cohort		SPARK
**Behavioral Measures**	Autistic	Non-autistic	SPARK ASD
PSQ-Total Score (n)<br>Mean (SD)<br>Median (Range)	n=41<br>1.73 (1.64)<br>1 (0-6)	n=49<br>0.61 (0.91)<br>0 (0-3)	n=205<br>1.29 (1.36)<br>1 (0-5)
RBQ-2A Total Score (n)<br>Mean (SD)<br>Median (Range)	n=96<br>1.93 (0.46)<br>2 (1.05-2.90)	n=80<br>1.30 (0.26)<br>1.25 (1.00-2.30)	n=210<br>1.84 (0.42)<br>1.85 (1-2.95)
RBQ-2A Factor 1 Score (n)<br>Mean (SD)<br>Median (Range)	n=96<br>1.79 (0.58)<br>1.66 (1.00-3.00)	n=80<br>1.25 (0.27)<br>1.16 (1.00-2.33)	n=210<br>1.61 (0.45)<br>1.5 (1-3)
RBQ-2A Factor 2 Score (n)<br>Mean (SD)<br>Median (Range)	n=96<br>2.02 (0.49)<br>2.10 (1.00-2.90)	n=80<br>1.32 (0.30)<br>1.27 (1.00-2.54)	n=210<br>1.99 (0.48)<br>2 (1-3)
Right-hand Finger Tapping (n)<br>Mean (SD)<br>Median (Range)	n=107<br>43.91 (9.74)<br>44.33 (6.33-66.33)	n=93<br>43.73 (9.53)<br>44.33 (17.33-62.33)	—
Left-hand Finger Tapping (n)<br>Mean (SD)<br>Median (Range)	n=107<br>41.62 (8.52)<br>42.00 (5.66-61.33)	n=93<br>41.41 (8.13)<br>41.66 (21.00-59.66)	—
**MRI Sample**			
eTIV% Left Caudate (n)<br>Mean (SD)<br>Median (Range)	n=111<br>0.22 (0.04)<br>0.22 (0.14-0.36)	n=94<br>0.22 (0.02)<br>0.22 (0.16-0.28)	—
eTIV% Right Caudate (n)<br>Mean (SD)<br>Median (Range)	n=111<br>0.23 (0.04)<br>0.22 (0.12-0.38)	n=94<br>0.22 (0.02)<br>0.22 (0.17-0.29)	—
Left Precentral Gyrus (n)<br>Mean (SD)<br>Median (Range)	n=111<br>2.63 (0.15)<br>2.65 (2.16-2.99)	n=91<br>2.66 (0.14)<br>2.67 (2.14-2.96)	—
Right Precentral Gyrus (n)<br>Mean (SD)<br>Median (Range)	n=111<br>2.61 (0.16)<br>2.61 (2.12-2.96)	n=91<br>2.62 (0.15)<br>2.63 (2.06-2.99)	—
eTIV% Left Putamen (n)	n=111	n=94	—

Mean (SD)<br>Median (Range)	0.32 (0.07)<br>0.32 (0.22-0.54)	0.31 (0.09)<br>0.33 (0.26-0.43)	
eTIV% Right Putamen (n)<br>Mean (SD)<br>Median (Range)	n=111<br>0.34 (0.07)<br>0.33 (0.24-0.53)	n=94<br>0.32 (0.09)<br>0.33 (0.28-0.46)	—
eTIV% Left Cerebellum (n)<br>Mean (SD)<br>Median (Range)	n=111<br>3.55 (0.65)<br>3.53 (2.85-5.01)	n=94<br>3.29 (0.92)<br>3.43 (2.60-4.21)	—
eTIV% Right Cerebellum (n)<br>Mean (SD)<br>Median (Range)	n=111<br>3.57 (0.42)<br>3.50 (2.81-4.97)	n=94<br>3.49 (0.33)<br>3.47 (2.59-4.27)	—

Note. Values are presented as mean (SD) and median (range). Sample sizes (n) reflect the number of participants with available data for each measure. ASD=autism spectrum disorder; eTIV=estimated total intracranial volume; MRI=magnetic resonance imaging; PSQ=Parkinsonism Screening Questionnaire; RBQ-2A=Repetitive Behaviors Questionnaire-2 Adult; SD=standard deviation; SPARK-Simons Powering Autism Research for Knowledge. Em dashes indicate that the measure was not available for the SPARK cohort.
